# Mapping the complex everyday challenges and needs of people with rheumatic disease and their surroundings using a multi‐actor approach

**DOI:** 10.1002/msc.1639

**Published:** 2022-04-27

**Authors:** Simone Harmsen, Joyce A. Nabuurs, Lotte F. Lehman de Lehnsfeld, Marjoleine G. van der Meij, Jacqueline E. W. Broerse, Carina A. C. M. Pittens

**Affiliations:** ^1^ Faculty of Earth and Life Sciences Athena Institute VU University Amsterdam The Netherlands; ^2^ Dutch Arthritis Society Amsterdam The Netherlands

**Keywords:** caregiving, patient experiences, rheumatology

## Abstract

**Introduction:**

This study aimed to gain insight into the real‐world complexity of the challenges experienced by patients, their significant others, care professionals and the work and education environment concerning rheumatic diseases as well as the interrelation between these challenges; it also aimed to prioritise the identified challenges.

**Method:**

Using the Dialog Model, 21 people with various rheumatic diseases, 24 care professionals, 9 significant others, and 3 education and work representatives were asked about rheumatic disease‐related challenges and needs in a series of focus groups and interviews. Data were inductively coded and analysed, resulting in a mind map thematically displaying the challenges. The mind map was translated into a survey, and respondents (*N* = 1802) prioritised themes and challenges.

**Results:**

Of the six identified themes, ‘physical complaints’ was prioritised the most, followed by ‘collaboration in healthcare’, ‘social and mental wellbeing’, ‘self‐management’, ‘information and options in healthcare’ and ‘work and education’. Challenges of people with rheumatic diseases appeared to be complexly interrelated. For instance, fatigue and pain affect everyday functioning, but can also heavily impact social and mental wellbeing. To facilitate support for these challenges, which many patients desire, patients and care professionals said that better collaboration between primary and secondary care professionals is needed. Additionally, patients felt that their experiential expertise deserves more acknowledgement from care professionals. Results were similar across different rheumatic diseases.

**Conclusion:**

Many patients desire more support to manage life with their disease. To facilitate this, collaboration and communication between healthcare professionals, and between healthcare professionals and individual patients, should be improved.

## 1 INTRODUCTION

Globally, musculoskeletal diseases, among which rheumatic diseases, put a significant burden on societies: they currently rank first among causes of years lived with disability (YLD), with an increase in YLD of 19.8% between 2010 and 2019 (Vos et al., 2020), causing lower quality of life (Branco et al., 2016; Sloot et al., 2016). Studies report impaired mental health (Branco et al., 2016; Kristiansen et al., 2012; Park et al., 2020), negative effects on social interactions (Sloot et al., 2016) and challenges within the healthcare system, such as insufficiently effective medication (Radawski et al., 2019) or limited attention being given to pain (Erwin et al., 2018).

Due to the diverse impacts of rheumatic diseases on a range of societal, physical, social and care aspects, there is an increasing interest in the needs and questions of patients. This has been inspired by studies in other disease areas which have shown that although patients' perceptions may overlap with those of professionals and researchers, they can also be substantially different (e.g. Broerse et al., 2010; da Silva et al., 2010; Tallon et al., 2000). In the area of rheumatic diseases, the objectives for collecting insights on patient perceptions have varied from improving care practices (e.g. Erwin et al., 2018) to building research agendas (e.g. Parsons et al., 2017; Radawski et al., 2019). Recent studies on the quality of life and needs of patients with rheumatic diseases mainly used quantitative surveys (e.g. Hunter & Riordan, 2014; Ingegnoli et al., 2020). While quantitative research has provided relevant insights into the prevalence of challenges such as pain and fatigue, it does not allow insights into the real‐world complexity behind survey responses. Additional qualitative research is essential to acknowledge and map contextualised experiences of patients. Various studies have taken such a qualitative approach but have explored needs within a specific care context (e.g. Erwin et al., 2018; van Eijk‐Hustings et al., 2013) or have aimed at research agenda creation (e.g. Verwoerd et al., 2021) and have not integrated perspectives of actors in their surroundings that provide their care and support. Non‐majority perspectives or conflicts in perspectives among various actors therefore remain hidden.

A recent review by Fairley et al. (2021) concluded that identification of patients' needs that are broader than healthcare is needed to improve person‐centred care. We argue that challenges are best investigated holistically to inform a broad architecture of solutions that address these challenges. As highlighted by Fairley et al. (2021), an exploration of the complexity of the life experiences and challenges of rheumatic disease patients is currently missing. Considering the challenges of those who closely interact with patients and are responsible for their care and support can provide a further base for the exploration of solutions. To the best of our knowledge, such a broad, multi‐actor perspective has not been performed or documented yet in the scholarly literature.

The current study, therefore, aims to gain insight into the real‐world complexity and variety of the daily life challenges experienced by patients and their family and friends, healthcare professionals and their work and education environment in relation to rheumatic diseases in the Netherlands and the interrelations between these challenges. These insights could provide a starting point for the development of a broad array of solutions to these challenges.

## 2 METHODS

This study was part of a larger action research project that aimed to develop strategic aims for the Dutch Arthritis Foundation (DAS). The first part of the project aimed to explore and prioritise the challenges experienced by patients and those who provide their care and support; we report on this part in this paper (hereafter: ‘this study’).

We based our approach on the principles of the Dialog Model (DM; Abma & Broerse, 2010). The DM is a transdisciplinary research approach to formulating and prioritising complex health‐related problems, that applies different principles to ensure successful multi‐actor consultations: active engagement of relevant actors, good social conditions, respect for experiential or practical knowledge, emergent and flexible design, and neutral and structuring process facilitation.

In the first phase of this study we explored the experienced challenges of patients and those who provide their care and support through online, creative focus group discussions (FGDs) and interviews. Such an open approach is essential to acknowledge the richness of real‐life experiences and the complexity of different actors' challenges. In the second phase, these challenges were ranked by a broad group of actors through a survey. The data were gathered between May and December 2020.

### 2.1 Patient and public involvement

In preparation for the overall project, we interviewed various employees of the DAS, healthcare professionals and researchers to get their advice on the design of the project and the actors that should be included. We also interviewed two patient representatives of the DAS to get their advice about the design and execution of the data collection activities during phase 1 of this study. The patient representatives were, together with other employees of the DAS, also part of an advisory board. During the first advisory board meeting, they reflected on the envisioned design of this study, and the actors that should be invited. During a second meeting, they reflected on the study's progress and on a concept version of the qualitative analysis.

### 2.2 Phase 1: Problem identification (May–October 2020)

#### 2.2.1 Participant selection

We aimed to include: (1) patients with a large variety of rheumatic diseases, and actors who (potentially) have an important role in providing (in)formal care and support for patients; (2) family and friends (hereafter ‘significant others’; 3) primary, secondary and tertiary healthcare professionals working with rheumatic disease patients, and (4) employment and education experts. Participants with rheumatic diseases and significant others were recruited through the DAS′ network and invitations on various (online) platforms (e.g. DAS website and network). We identified healthcare, work and education professionals with rheumatic diseases expertise through an Internet search. We invited them to participate by email and telephone, either personally or through an open invitation to organisations or departments. Using Google forms, patients, significant others and professionals signed up directly for an FGD or group interview. We use the term ‘FGD’ for sessions of four to seven participants, and ‘group interview’ for sessions two to three participants. One patient was not able to participate online, and we, therefore, interviewed them by telephone. Because a limited number of significant others signed up for the FGDs, we conducted one additional interview by telephone after the FGDs. After consultation with the advisory board, we decided to conduct additional interviews with two GPs and a psychologist, as these professions had not yet been represented in the two FGDs with healthcare professionals. In total phase 1 included 57 participants.

#### 2.2.2 Data collection

Due to 2020s COVID‐19 restrictions, all data collection (Table 1) occurred online. We used Zoom for a series of two‐hour FGDs and group interviews, guided by two facilitators. Table 2 shows the set‐up of all FGDs and (group) interviews, for which we used Padlet (an online application for digital Post‐it brainstorming) as a conversation tool. We provided all participants with extensive technical support to allow full participation. All Padlet boards were saved, and sessions were audio and video recorded after consent was given.

**TABLE 1:** Participants, phase 1

Actor group	Actor subtype	Number of participants
Patients		
	Osteoarthritis	7
	Fibromyalgia	7
	Gout	3
	Rheumatoid arthritis	4
	Polymyalgia rheumatica	2
	Ankylosing spondylitis	2
	Other rheumatic diseases	7
	Total (in 2 FGDs, 5 group interviews and 1 individual interview)	21^a^
Significant others		
	Family members and friends of people with rheumatic disease. (In 3 group interviews and 1 individual interview)	9
Primary care professionals		
	Sports coach	1
	Nutritionist	1
	Physiotherapist	4
	Occupational therapist	1
	General practitioner	2
	Psychologist	1
	Exercise therapist	2
	Total (in 1 FGD and 3 individual interviews)	12
Secondary and tertiary care professionals		
	(Child) rheumatologist	2
	Nurse counsellor	1
	Rheumatology nurse	1
	Health scientist/coach	1
	Rehabilitation doctor	3
	Hospital (child) physiotherapist	3
	Physician assistant	1
	Total (in 1 FGD)	12
Work and education representatives		
	Representatives of organisations who help young people with chronic diseases to find employment^b^	2
	Researcher studying participation in the labour market^b^	1
	Total (in 1 group interview)	3
**Total**		**57**

^a^ Because most patients had multiple diseases, the total number of diseases does not equal the total number of patients.

^b^ As employers or education facilities did not respond invitations to participate, other experts/representatives who could provide insights on the impact of rheumatic diseases on work and education in general, and for employers and education facilities specifically, were included.

**TABLE 2:** The set‐up of all (group) interviews and FGDs

Element	Description
Session preparation	Several days before their session, patients receive a preparatory booklet containing creative preparatory exercises (cf. Mattelmäki, 2006; Sleeswijk Visser, 2009). This preparation is done to allow for a shorter, less burdensome focus group or interview, by sensitising participants to the topic beforehand.
	Significant others receive preparatory questions to deliberate on to sensitise them to the topic.
Session part 1	Exploration of challenges people experienced while living with rheumatic diseases or taking care of people with rheumatic diseases.
1a^a^	Participants write down their experienced challenges on Post‐its in the Padlet application. Simultaneously, facilitator 1 organizes their challenges in clusters. Participants can see each other's contributions on the Padlet board.
1b^a^	The challenges are discussed, guided by facilitator 2. The facilitator asks participants to elaborate on their posted answers, aiming to gain deeper insights into the relationships between and causes and impacts of these challenges. Meanwhile, facilitator 1 adds new insights from the discussion to the Padlet board, which all participants can see.
1c	Participants are asked to mark the challenges they prioritised.
Session part 2	Exploration of participants' need to overcome the challenges identified in part 1.
2a^a^	Participants write down their needs on Post‐its in the Padlet application.
2b^a^	The needs are discussed, guided by facilitator 2. Meanwhile, facilitator 1 adds new insights from the discussion to the Padlet board, which all participants can see.
2c^a^	Participants are asked to mark the challenges they prioritised.

^a^ During the telephone interviews, no Padlet board was used, but interviewees were asked about the same topics.

#### 2.2.3 Analysis

Directly after initiating data collection, we started **open coding** the Padlets, field notes and audio recordings. We inductively coded participants' experienced challenges (Strauss & Corbin, 1990) using Atlas.ti 9. Expressed needs were translated into challenges by formulating them as something that is currently missing. Through iterative **open** and **axial coding** (Strauss & Corbin, 1990), codes were compared, refined and connected to understand different components of the challenges and the relationships between them. Through **selective** coding (Strauss & Corbin, 1990), related challenges were clustered into themes using the ‘networks’ function of Atlas.ti to create a mind map of challenges. Open, axial and selective coding was done through a continuous iterative process. By ongoing analysis of the data directly after each FGD or (group) interview, we gradually induced predominant themes for the challenges mentioned by multiple actors. This led to purposeful data collection during subsequent sessions by prompting with the previously identified themes in mind (Birks & Mills, 2015), enabling us to gradually improve our understanding of the (causal) relationships between challenges. Authors SH, JN, LLL, MM and CP contributed to coding through frequent conversations, negotiations and decision‐making regarding the final set of themes.

### 2.3 Phase 2: Priority setting

#### 2.3.1 Survey design

To gain insights into the way patients and other actors prioritised the identified challenges and needs, we designed a survey using Qualtrics online software (detailed in appendix A). The previously induced themes were translated into ‘topics’ and grouped in clusters. To limit the number of topics in the survey, various highly interconnected themes were merged. The survey asked respondents to suggest what “more attention should be paid to…”, asking them to choose two topics per cluster, wherein ranking something first meant ‘needs the most attention’ and ranking something second meant ‘needs the next (second) level of attention’.

#### 2.3.2 Survey distribution

The survey was open to all interested individuals and was distributed among the patient panel of the DAS, the IPSOS rheumatic disease patient panel, a large group of researchers working on rheumatic diseases and all participants in phase 1. It was also dispersed through the DAS's social media and newsletter, reaching donors, members and other actors affiliated with the DAS.

#### 2.3.3 Analysis of survey

Survey data were transferred from Qualtrics to SPSS (version 26). For the analysis, we followed a descriptive statistics methodology similar to the one used by Broerse et al. (2010). Priorities given to themes were allocated points (#1 priority: two points, #2 priority: one point). For example, if the topic pain was prioritised 150 times; 50 times as #1 priority and 100 times as #2 priority, the prioritisation score was 50 × 2 + 100 × 1 = 200. Higher priority scores resulted in a higher ranking. We stratified our results for patients, significant others, healthcare professionals and ‘other’, and for all patient groups bigger than *N* = 100.

## 3 RESULTS

In this section, we first present the predominant challenges induced by our analysis. We discuss the relationships between the challenges faced by patients, significant others, healthcare professionals, and employers and schools, illustrated by sections of the mind map. Subsequently, we present how various actors prioritised these challenges in the survey. The illustrative conversation extracts were translated from Dutch to English by the authors and slightly adjusted for readability purposes.

### 3.1 CHALLENGES

**FIGURE 1:**
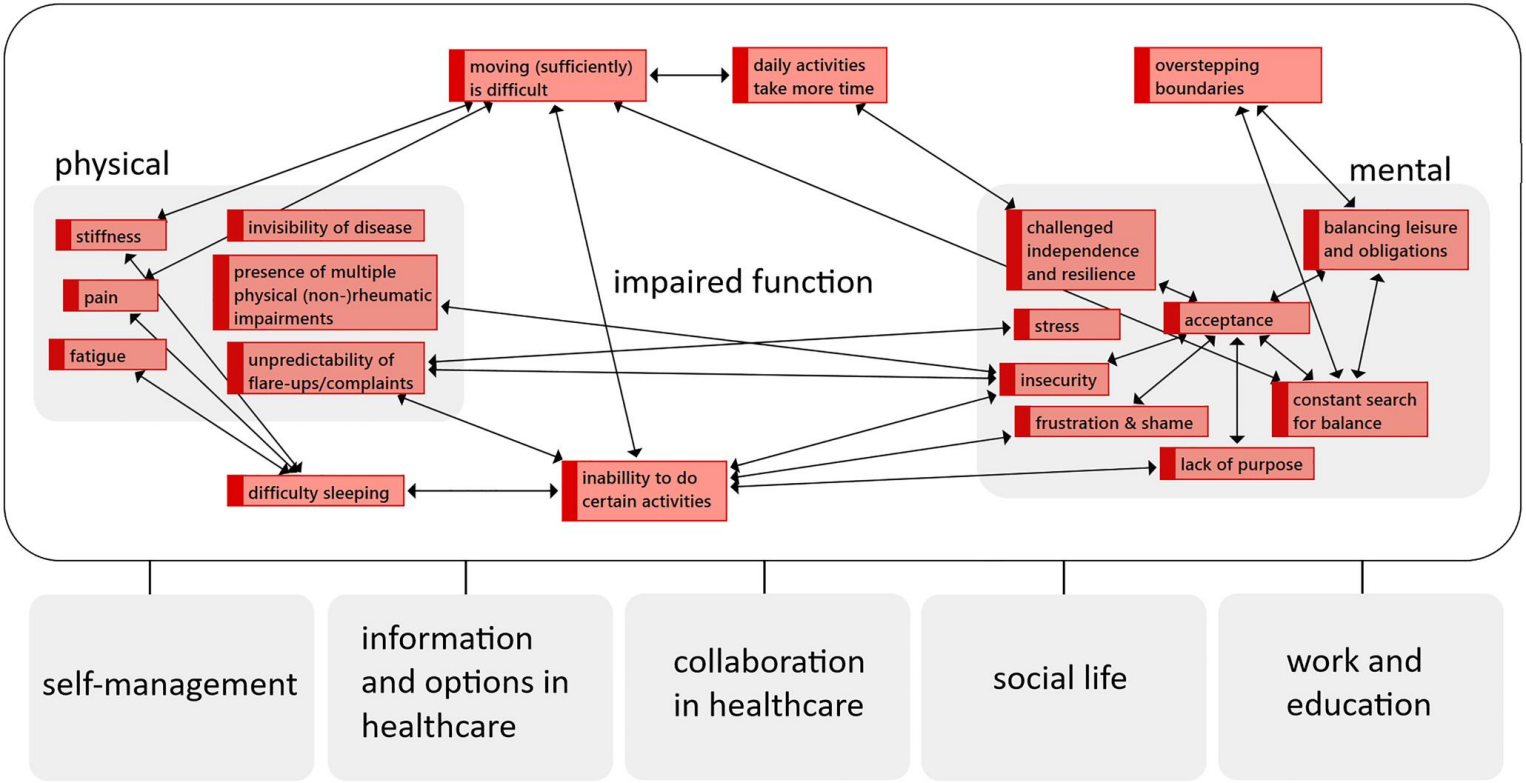
Relationships between physical and mental challenges and their effects on the way people function. These challenges lead to challenges in other parts of life, which we clustered into five (interconnected) themes

We identified eight major, highly interrelated themes of challenges (Table 3 and Figure 1).

**TABLE 3:** The eight major themes of the identified challenges

Theme	Description	Comprises challenges experienced by
Physical challenges	Physical symptoms of the rheumatic disease and the various ways these present themselves	Patients
Mental challenges	Challenges regarding patients' mood and thinking	Patients
Impaired function	The effect of physical and mental challenges on patients' behaviour and the functioning of the body	Patients
The challenges of patients in the rows above lead to challenges in other parts of life, in their relationships with other actors:		
Self‐management	Challenges regarding patients' ability to manage daily life with their rheumatic disease while aiming to manage or minimise the rheumatic disease	Patients and healthcare professionals
Information and options in healthcare	Challenges regarding the availability of information and (care) options in healthcare	Patients and healthcare professionals
Collaboration in healthcare	Challenges regarding communication and collaboration between healthcare professionals, and between patients and healthcare professionals	Patients and healthcare professionals
Social life	Challenges regarding relationships with other people	Patients and significant others
Work and education	Challenges regarding employment and education	Patients, employers and schools

Patients generally expressed similar challenges; differences between patients appeared less dependent on the type of rheumatic disease and more on their life and disease phase. Challenges seemed more profound during flare‐ups (i.e. for AS and RA) and around time of diagnosis. Relatively young people articulated more challenges regarding diagnosis and participation in social life, work and school, while patients of older age (above approximately 70) more often struggled with multiple rheumatic diseases or with different additional impairments.

As Figure 1 displays, certain themes are highly interconnected, and we will therefore discuss them in three parts.

#### 3.1.1 Physical and mental challenges and impaired function

Figure 1 shows the physical challenges that patients experienced, their impairing effect on function and the resulting mental challenges. The effects of physical challenges on functioning in daily life were often mentioned as part, or the root cause, of another challenge and are therefore discussed within the themes of physical and mental challenges. Some challenges were also recognized by significant others and healthcare professionals.

##### Physical challenges

During the FGDs, most patients mentioned ‘pain’ and ‘stiffness’ as common physical complaints. The most mentioned consequence of these was being less able to move, making certain activities difficult or impossible, such as lifting things at work or hobbies like rowing, biking or gardening. Many patients also experienced (excessive) fatigue, generally and/or after activities.

For many patients, the unpredictability and invisibility of symptoms were particularly challenging as they influence social life (discussed later) and scheduling. Also, pinpointing and maintaining physical limits was reported to be difficult, because negative physical effects are often delayed. Combined with mental challenges, this causes patients to push their limits.

Many patients said they struggled to move (sufficiently). Moving aids stiffness and overall health, but moving can also cause or worsen complaints. Many patients struggled to find the right form and amount of exercise. This can lead to daily dilemmas, that is exercising while in pain to alleviate pain, and movement being both the remedy for and the cause of stiffness.

“Sports feel like a vicious circle. For example, you should exercise more, but if you cannot exercise – if already your abdominal exercises cause pain in your toe, for example – you do not do it anymore. And then it worsens. You get into a vicious circle.” [P1, FGD2]

“Sure I am stiff, but when I have done a forest walk, I am completely flexible again. When I am in the car [going] back home, [and] when I am home I am already stiff again. So I have both stiffness because of not moving, and stiffness because of moving.” [P2, FGD1]

Similarly, several patients said that lying still in bed increased stiffness and pain, thereby disturbing sleep and further increasing fatigue.

##### Mental challenges

Patients reported that physical challenges and their impact on functioning caused various mental challenges. Many voiced a constant search for balance in life, not only regarding the (amount of) physical activities and sleep they had but also regarding balancing their limited energy between leisure and obligations. Leisure activities improve mental wellbeing but may worsen symptoms. Many of the patients said they did certain activities but accepted that they would ‘pay the price’ later.

Some found it difficult to accept their disease and the resulting limitations on their life. Challenges like the constant search for balance, increased dependence on others, and flare‐ups’ unpredictability caused frustration, insecurity and stress, and sometimes even shame.

“Perhaps I do not show enough what bothers me. So, people do not really know what is wrong with me. And I sometimes find it annoying to say that. Because you have been able to manage your entire life yourself; have been an independent person. And then it is sometimes difficult not to be able to do things.” [P3, FGD5]

Mental challenges link to challenges in social life, which we will discuss later.

#### 3.1.2 Challenges regarding self‐management and information, options and collaboration in healthcare

**FIGURE 2:**
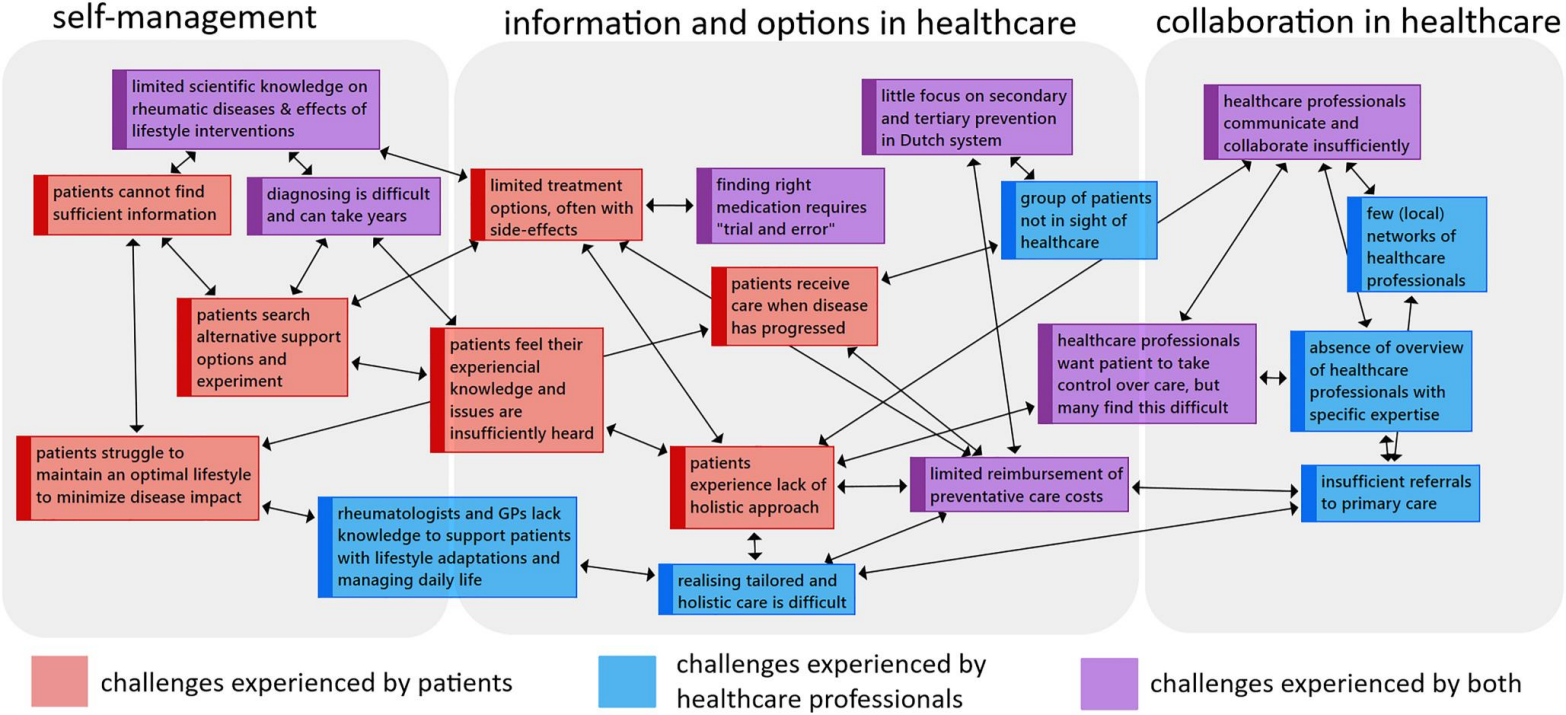
Challenges experienced by patients and healthcare professionals regarding self‐management and information, options and collaboration in healthcare

Figure 2 illustrates the main challenges that were faced by patients and healthcare professionals.

##### Self‐management

Almost all patients said that they constantly looked for ways to manage and balance their life with their rheumatic disease via food, exercise and the optimal scheduling of activities. Many expressed a need for support in this self‐management.

“There are medical solutions, but of course there are also a lot of things that are less invasive, and where you still have the feeling that you have more control over your own body.” [P3, FGD5]

The importance of maintaining the most helpful lifestyle and the difficulty maintaining it were recurring topics for patients and primary care professionals. Most patients found it difficult to find sufficient information on their disease and how to manage it. Some patients and healthcare professionals feared that being unable to maintain the most appropriate lifestyle could increase the burden of the disease on the individual and the healthcare system. GPs and some secondary care professionals recognized patients' need for active support concerning lifestyle but said they lack the relevant knowledge and skills to offer this because scientific knowledge about the influence of lifestyle (mainly exercise and diet) on various rheumatic diseases is currently absent. Primary care professionals, on the other hand, emphasised the importance of general health and lifestyle education:

“What people are looking for is some basic information. I think that we as primary care providers, who work very much from lifestyle, are very much in agreement that in the education of people and children, young adults there should be more space for taking good care of oneself.” [Dietician, primary care]

Healthcare professionals, especially primary healthcare professionals, believed that more frequent referrals to primary care professionals with relevant expertise is important to aid patients with self‐management. However, this is experienced as difficult, as we will discuss in the ‘collaboration in healthcare’ section. The GPs also noted that many patients with mild and/or early symptoms can sufficiently manage life with the disease by themselves, and do not seek healthcare.

##### Information and options in healthcare

The patients noted the challenges that exist regarding the availability of certain types of information and care. The healthcare professionals provided insights into the reasons why diagnoses and the allocation of adequate care can be difficult.

The limited scientific knowledge available on rheumatic diseases constrains early diagnosis. Many of the consulted patients had waited years for a (correct) diagnosis, which GPs and some secondary/tertiary care professionals recognized. For many patients, the long period of ‘not knowing’, plus a lack of information on the disease (prognosis) can greatly impact mental health, causing insecurity and frustration. Many patients also said that obtaining adequate care could be challenging. Most rheumatic diseases are chronic, and treatment options are often limited to pain medication, biologicals and/or joint replacement (for osteoarthritis). Various patients and a few secondary/tertiary professionals said that due to limited scientific knowledge, finding the right medication requires ‘trial and error’.

“Beforehand, we do not know well which treatment works for which patient. First you go to treatment one, and if that does not work you go to treatment two, and if that does not work you go to treatment three. Then another year has passed, while in the meantime damage is occurring.” [Physician assistant, secondary care]

The lack of (adequate) medication can cause damage and negative side‐effects like fatigue or impaired fertility. Given the chronic nature of their complaints, many of the patients desired more care and support with self‐management, mental health and managing daily life. While care professionals said that primary care could take on this task, both patients and healthcare professionals saw limited insurance reimbursement as a problem.

“[I am] not having access to physiotherapy reimbursement because I am not ‘bad’ enough yet, because I can still work. But shouldn't attention be paid to prevention, so it does not get that bad?” [P4, FGD6]

Limited reimbursement was attributed to there being little focus on secondary and tertiary prevention within the Dutch system. Healthcare professionals noted that this also caused many people with rheumatic disease to remain under the radar of care professionals. Both patients and healthcare professionals generally desired a more holistic approach to care where patients' needs and treatment goals form the starting point, and various care and treatment options are explored. Some patients said they want to be seen as a person rather than ‘an illness’. Although patients might not always feel acknowledged as such, our FGDs with professionals indicate that they are aware of patients' need for holistic care. However, they appear insufficiently able to respond due to limited reimbursement and limited collaboration (discussed later). Finally, several patients expressed an interest in alternative medicine (i.e. orthomolecular therapy) and said they valued health professionals' support while exploring it. None of the healthcare professionals spoke about alternative medicine during the FGDs.

##### Collaboration in healthcare

Patients, significant others and healthcare professionals all mentioned various challenges that hinder collaboration and communication in healthcare and that eventually constrain holistic care provision. Furthermore, they seemed to envision patients' role in healthcare differently.

A few patients and several significant others described their experience of patients having to actively take charge of the communication and collaboration between healthcare professionals. They had experienced frustration caused by bureaucracy and the separation of various specialisms and therapists. Those who had sought additional (primary) care also had trouble finding rheumatic diseases experts, for example specialised dieticians.

Many of the healthcare professionals had similar difficulties locating colleagues and making referrals to them. Almost all of the secondary care professionals and GPs said they lacked an overview of (primary) healthcare professionals with specific rheumatic disease expertise. Primary care professionals affirmed that secondary care professionals and GPs could not always find them.

“They are not visible, people in primary care that do have that expertise. That is a problem, I think. I think that it is also automatically thought ‘then I just keep it within secondary care’. [So] the knowledge is not there, they are not clearly visible, accessible; and the financing.” [Physical therapist, secondary care]

However, several primary care professionals in the FGDs said secondary and tertiary care professionals ‘pathologised’ patients, and that for many patients, more general, basic care could be sufficient.

“It appears that rheumatologists seem to have a limited understanding that some patients can see a basic therapist, while others need specialist care. Let's offer the total range of rheumatism patients, let them receive the care that is necessary, but also do not commit to pathologising too much." [Physical therapist, primary care]

Professionals often discussed the role of the patient in communication and initiating healthcare. This is related to patients' self‐management, but also the collaboration between patients and professionals. Various professionals mentioned that patients' capacity to take ownership of their health and care is an important determinant for healthcare outcomes, and described how health literacy largely influences this.

“As a patient, being in control of your life with rheumatic disease is very important, and having health literacy is a factor in that. Like, when do I sound the alarm, when should I take action myself? When should I contact my practitioner if there is something wrong, and who should I contact?” [Physician assistant, secondary care]

While many professionals would like patients to take a more active role in organising their healthcare, they also struggled with how to deal with patients who are less capable of doing this. Interestingly, some patients said they needed more help to find caregivers or therapeutical options but wanted the burden of taking responsibility to be reduced. Other patients wanted more say in decision‐making: they wanted more of their questions and suggestions to be heard. Some felt their experiential knowledge and experimentation were not always taken seriously.

“Sometimes you know more than experts, so openness to sparring with healthcare professionals is nice, for example about the influence of nutrition.” [P5, FGD6]

Some patients added that they felt their disease complaints had not always been given much weight, especially by their GP. Two GPs commented that because they see many patients with ‘vague’ complaints, often at early‐stage rheumatic disease, diagnosing can be difficult and this may make patients feel unheard.

#### 3.1.3 Challenges in social life and in work and education

A mind map displaying the challenges experienced in social life and in work and education is available under supplementary materials.

##### Social life

Patients often discussed the influence their rheumatic disease had on their social life. Many said they were generally hesitant to speak openly about their disease. Fear of misunderstanding, ‘burdening’ others and shame appear to play a role. Similar challenges were expressed by significant others, who sometimes struggled with how to deal with their loved ones' condition. Many reported being misunderstood by outsiders. Many patients said they received negative reactions to their limited abilities or (last minute) cancellations as a result of unpredicted disease flare‐ups. This was said to occur because people generally underestimate the consequences and severity of rheumatic diseases, which might be caused by a general lack of knowledge about rheumatic diseases. They mentioned that rheumatic diseases have little societal visibility, as they are often not physically visible and patients stay at home during flare‐ups.

“What I have come across a lot in my work, is colleagues say ‘things are going well today, are they not?’. What I found difficult about that: when things are not going well, and I could not go to work, I was homesick. So, they only see you on the days that you are functioning.” [P6, FGD4]

This misunderstanding impacted the mental health of many participants, and resulting social pressure could (especially among younger people) impact physical health because it made them overstep their physical limits. Several significant others had seen patients hide or downplay their disease in front of others. Some significant others said that they could sometimes inadvertently push their loved ones' limits, because it could be difficult for them to balance assisting with allowing independence. Some of them went on to say that this sometimes made them feel guilty and/or powerless. Spouses especially articulated that their partner's disease could put a strain on them and their relationship.

“There are so many things we can no longer do together. My partner feels guilty about that. [Usually] I say that that is not necessary, but it still puts a strain on our relationship.” [Significant other, FGD 8]

Some spouses described having to do more tasks due to their partner's disease, and a few patients and spouses mentioned that the illness impacted their intimacy, sexuality or ability to have children negatively.

##### Work and education

Various patients reported that their disease impacted employment. Two specifically confirmed that there were challenges regarding education. Work and education representatives also explored challenges for employers and schools concerning students or employees with rheumatic diseases.

Several participants of various ages and with different rheumatic diseases were unemployed or were forced to work part‐time due to their disease. Some had been (unwillingly) declared to be partially or permanently incapacitated regarding work by the Employed Person's Insurance Administration Agency (UWV). Some consequently suffered financial and mental effects. Employed or studying patients found organisations insufficiently accommodating; conditions, such as desks or working hours, were not always adapted for their needs. Some faced negative reactions from colleagues or peers, especially young patients whom people deemed ‘young and vital’.

Work and education representatives said employers can face practical, communicational and financial challenges: jobs might not be adaptable, or adaptations are costly. They also noted that communication between patients and employers about the rheumatic disease and what it is (im)possible for patients to do can be difficult – something patients also mentioned. Patients can be hesitant to discuss their disease, fearing negative consequences, and employers are bound by privacy laws.

### 3.2 Priority setting

#### 3.2.1 Respondents

The initial number of respondents to the survey was 2256, though only 1802 respondents completed the survey. Table 4 summarises respondents' socio‐demographic characteristics (extended overview in appendix B). For the purpose of this study, we focussed on patients, significant others, and healthcare professionals; all other respondents were grouped under ‘other’. The survey was not (identifiably) filled in by people from an employer or education perspective. Out of all respondents, 83.4% were patients; 51.3% of these had OA, 39.7% had RA, 29.1% fibromyalgia, 15.0% AS and 34.5% had another rheumatic disease (some patients had multiple diseases).

**TABLE 4:** Socio‐demographic characteristics of respondents; *N* = 1802

Variable	Frequency	%
**Role or link with rheumatic diseases:**		
Patient	1503	83.4
Significant others	54	3.0
Healthcare professional	79	4.4
Other	166	9.2
**Gender** **:**		
Male	330	18.3
Female	1469	81.5
**Age:**		
18–25 years	26	1.4
25–35 years	98	5.4
35–50 years	352	19.5
50–65 years	822	45.6
65–80 years	476	26.4
>80 years	28	1.6

#### 3.2.2 Prioritisation of themes

**FIGURE 3:**
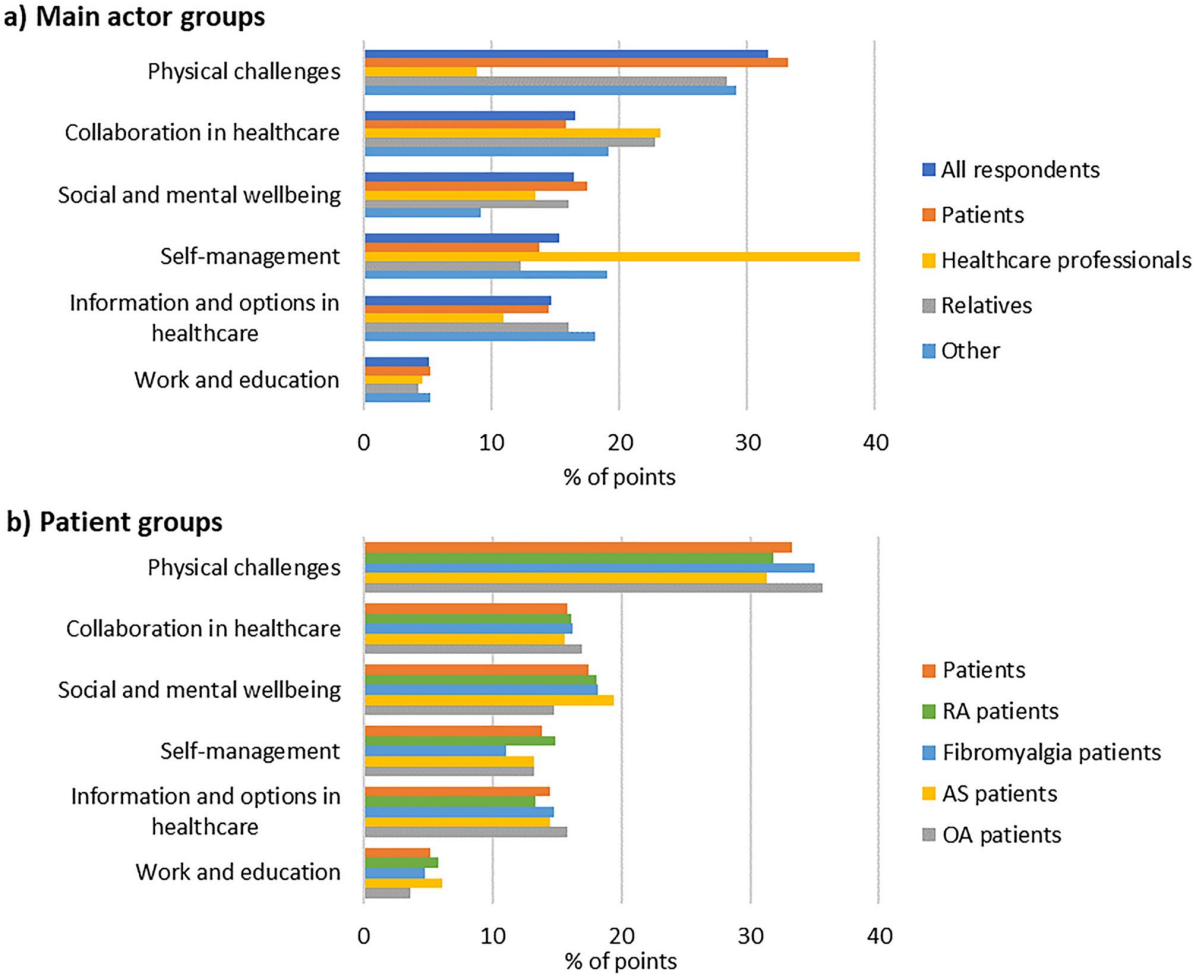
Prioritisation of themes by various (a) main actor groups and (b) patient groups. The percentage indicates the proportion of points allocated to the theme, relative to the total number of points allocated to all themes

Figure 3 shows the topics and themes ranked in order of the number of points they received. Displayed is the percentage of points given to each theme, relative to the total number of points allocated to the themes.

Generally, themes were ranked similarly by the various actor and patient groups. Although challenges regarding work and education were prominent in the FGDs, few survey respondents prioritised these. Collaboration in healthcare, information and options in healthcare, and social and mental wellbeing were prioritised very similarly. The prioritizations of physical challenges and self‐management are the most prominent. Physical challenges were ranked first by all respondent groups except healthcare professionals, who ranked them fifth. Healthcare professionals prioritised self‐management most often, giving it 38.8% of the points, while this was ranked only fifth by patients and significant others.

#### 3.2.3 Prioritisation of topics

Table 5 shows an overview of the prioritisation of themes and topics by various respondent groups.

**TABLE 5:** The ranking of themes and topics, stratified for various survey respondent groups. (For topics, the percentage is the proportion of points allocated to the topic, relative to the total number of points allocated to all topics within a theme. For themes, the percentage indicates the proportion of points allocated to the theme, relative to the total number of points allocated to all themes. Rank 1 and 2 are indicated by *green or yellow highlight respectively*.)

Respondent group (% of points) Themes and topics		All respondents	Patients	OA patients	AS patients	Fibromyalgia patients	RA patients	Significant others	Healthcare professionals	Other
**1 Theme: Physical challenges**		**31.6**	**33.2**	**35.6**	**31.3**	**35.0**	**31.8**	**28.4**	**8.9**	**29.1**
	a. Fatigue	**26.5**	**26.9**	*24.4*	**32.1**	**25.5**	**28.8**	22.2	**40.5**	17.7
	b. Pain	*25.3*	*24.7*	**30.3**	16.6	*24.3*	*22.3*	**30.9**	14.8	**34.3**
	c. Invisibility of many rheumatic diseases and complaints	18.9	18.9	17.4	*20.0*	21.7	18.4	*23.5*	12.2	*20.3*
	d. Unpredictability of complaints	15.1	15.7	13.6	16.1	14.0	16.8	9.3	14.8	12.0
	e. Presence of several physical complaints	14.2	13.8	14.3	15.1	14.6	13.7	14.2	*17.7*	15.7
**2 Theme: Collaboration in healthcare**		**16.6**	**15.8**	**16.9**	**15.6**	**16.2**	**16.1**	**22.8**	**23.2**	**19.2**
	a. (Local) network of primary and secondary healthcare workers	**28.5**	**27.6**	**26.4**	**29.0**	*24.8*	**28.6**	**24.7**	**40.1**	**32.1**
	b. Acknowledging the experiential expertise of patients	*19.6*	*20.5*	20.6	*20.7*	22.5	*21.4*	20.4	4.6	17.7
	c. Findability of specialised primary healthcare workers with expertise in rheumatic diseases	19.1	18.6	18.7	18.5	15.3	18.3	*22.8*	24.9	18.9
	d. Patients often feel they are not being taken seriously by doctors or that doctors think they know what is best for the patients	18.4	19.8	*22.1*	18.4	**28.1**	16.2	19.1	5.1	11.4
	e. The control of patients over their own healthcare	14.5	13.4	12.5	13.3	9.3	15.5	13.0	*25.3*	*19.9*
**3 Theme: Social and mental wellbeing**		**16.5**	**17.5**	**14.8**	**19.4**	**18.2**	**18.1**	**16.0**	**13.5**	**9.2**
	a. Constant search for balance in life: between ‘doing’ and ‘not doing’ and between things ‘I have to do’ and things ‘I like to do’	**30.9**	**31.9**	**31.3**	**34.4**	**34.9**	**30.8**	**25.9**	**34.2**	**21.7**
	b. Acceptance of rheumatic disease and its influence on life	*18.1*	*18.4*	*18.4*	*17.9*	14.8	*19.8*	15.4	*21.5*	15.3
	c. Misunderstanding from outsiders due to limitations	13.6	14.0	14.4	12.6	*16.8*	13.3	*25.4*	7.2	12.4
	d. The uncertainty a rheumatic disease can cause (i.e. due to a lack of information and delayed diagnosis)	10.5	10.4	12.1	9.3	9.8	8.5	6.8	9.3	12.9
	e. Awareness of rheumatic diseases and their consequences among the Dutch public	8.0	6.8	6.5	5.8	4.8	7.9	11.7	5.9	*18.1*
	f. (mental) impact of rheumatic diseases on close significant others	7.8	7.5	6.7	7.6	8.3	8.3	13.6	7.2	8.4
	g. Struggling to find meaning in life, because of lack/loss of work and inability to do other daily activities	5.3	5.1	5.2	5.2	4.7	4.9	3.1	8.0	6.6
	h. Shame and taboo around having a rheumatic disease	3.2	3.1	3.0	4.1	3.4	3.2	5.7	2.1	3.8
	i. Effects of rheumatic diseases on intimacy and sexual relations	2.6	2.7	2.3	3.1	2.4	3.4	2.5	4.6	0.8
**4 Theme: Self‐management**		**15.3**	**13.8**	**13.2**	**13.2**	**11.1**	**14.9**	**12.3**	**38.8**	**19.1**
	a. Influence of lifestyle on several rheumatic diseases	**23.6**	**23.1**	**21.9**	**24.4**	*22.1*	**24.6**	22.2	**30.8**	**24.7**
	b. Information on factors relating to effective lifestyle changes, such as nutrition and correct movement	*22.3*	*22.1*	*21.6*	18.8	17.8	*24.5*	*24.1*	*24.5*	*22.1*
	c. Actively supporting patients to optimise their lifestyle	20.4	19.6	20.8	21.2	21.9	19.7	**25.9**	21.5	**24.7**
	d. Actively supporting patients in living their everyday life with a rheumatic disease, for example, scheduling of activities and mental support	19.4	20.5	19.7	*23.3*	**24.9**	18.0	14.2	14.3	13.5
	e. Information regarding different rheumatic diseases and treatments	14.4	14.7	16.1	12.3	13.3	13.3	13.6	8.9	15.1
**5 Theme: Information and options in healthcare**		**14.7**	**14.5**	**15.8**	**14.5**	**14.8**	**13.3**	**16.0**	**11.0**	**18.1**
	a. Correct and fast diagnosis of rheumatic diseases	**22.9**	**22.4**	**22.9**	*21.2*	20.7	**21.1**	*21.6*	12.7	**33.1**
	b. Reimbursement of primary healthcare costs such as fees for physiotherapists, nutrition coaches and psychologists	*20.0*	*21.9*	**22.9**	**26.5**	**25.9**	*19.4*	9.9	*16.9*	7.8
	c. More holistic and multidisciplinary healthcare	19.8	19.1	18.8	20.1	*22.2*	18.1	**25.3**	**32.1**	*18.5*
	d. Mental health and support in learning to live with the rheumatic disease	12.4	12.8	11.5	11.6	13.7	13.1	11.1	11.8	8.6
	e. Side‐effects of medication	9.8	10.5	9.8	8.4	6.6	15.6	9.9	4.6	6.6
	f. Alternative treatments such as homoeopathy, acupuncture and orthomolecular therapy	7.8	8.4	9.2	6.4	8.3	7.8	8.6	1.3	5.0
	g. Prevention	3.6	2.4	2.9	2.4	1.4	2.4	9.3	5.1	12.7
	h. Lifestyle interventions	3.6	2.6	2.1	3.4	1.2	2.6	4.3	15.6	7.6
**6 Theme: Work and education**		**5.1**	**5.2**	**3.6**	**6.1**	**4.8**	**5.8**	**4.3**	**4.6**	**5.2**
	a. Undesired inability to work	**29.2**	**30.3**	**28.8**	**31.6**	**30.8**	**28.9**	19.8	21.5	*26.3*
	b. Attitude and flexibility of employers/educational institutes; thinking in terms of possibilities	*22.1*	20.2	20.8	*21.2*	*19.2*	21.0	**32.7**	*29.1*	**32.1**
	c. Communication between patients and employers/supervisors regarding their rheumatic disease, what it is possible for them to do and their needs regarding work	21.3	*20.7*	*21.1*	18.4	18.9	*21.8*	*21.0*	**34.2**	8.6
	d. Earning less or not enough as a result of the rheumatic disease	10.8	12.0	11.4	13.8	13.4	11.5	4.3	5.9	4.2
	e. Misunderstanding of colleagues or peers because of disabilities	8.5	8.5	10.1	6.5	10.4	8.8	14.2	3.8	8.0
	f. Difficulties finding a job	8.2	8.3	7.7	8.6	7.2	8.0	8.0	5.5	8.6

##### Patients

Patient groups prioritised most topics quite similarly. Fibromyalgia patients' priorities differed the most, though only minimally. Within ‘*physical challenges*’, patients ranked fatigue and pain first, followed by invisibility.

Within ‘*collaboration in healthcare*’, many patients prioritised option a, ‘(Local) network of primary and secondary healthcare workers’. During the FGDs this was not explicitly identified by patients as a need; however, they did struggle to find healthcare professionals and also mentioned communication and referral between professionals: this survey topic might have been seen as a solution to those challenges. Two topics have some overlap: ‘acknowledging the experiential expertise of patients’ and ‘patients often feel they are not being taken seriously by doctors or that doctors think they know what is best for patients’. Interestingly, these topics were ranked second and third by the overall patient population, indicating that patient–doctor communication might currently be unsatisfactory for many patients. OA and fibromyalgia patients prioritised the topic of being taken seriously over the topic of patients' expertise. Our qualitative results indicate that this could be because fibromyalgia is not universally recognized as a disease, and many patients experienced having to ‘fight’ to be taken seriously and ultimately be diagnosed. For OA, this may be because OA was said to sometimes be discarded as ‘just part of ageing’.

The constant search for balance in life re‐emerged in all patient FGDs and was ranked highest by all patient groups in the theme ‘*social and mental wellbeing*’. This challenge could be a ‘meta‐challenge’: it is the result of many other daily challenges and is therefore prioritised very often. A correct and fast diagnosis was prioritised by many patients. Patients voiced in the FGDs that delayed diagnosis was frustrating and caused insecurity, greatly impacting their lives. Reimbursement of primary care costs was prioritised often as well.

##### Healthcare professionals

Healthcare professionals' and patients' priorities differed on various topics. The qualitative results combined with those shown in Table 5 provide clues about why ‘*self‐management*’ was prioritised by healthcare professionals. During FGDs, professionals stressed how important patients having ‘health literacy’ and ‘control over their health’ was for rheumatic disease progression/improvement. They often used similar words to indicate the importance of patients taking responsibility for organising their healthcare. This definition of ‘self‐management’ possibly influenced how professionals ranked the theme ‘*self‐management*’. Indeed, the topic ‘the control of patients over their own healthcare’, in the theme ‘*collaboration in healthcare*’, was prioritised often by healthcare professionals. Within the theme ‘*self‐management*’, healthcare professionals (like patients and others) prioritised ‘the influence of lifestyle on several rheumatic diseases’. Yet in the theme ‘*information and options in healthcare*’, the related topics ‘lifestyle interventions’ and ‘prevention’ were rarely prioritised. Some professionals said the absence of scientific evidence elucidating the influence of lifestyle on rheumatic diseases made giving lifestyle advice difficult. Survey respondents might therefore have reasoned that scientific evidence is a key challenge that needs resolving before ‘lifestyle interventions’ become possible.

Notably, within ‘*physical challenges*’, ‘presence of several physical complaints’ was prioritised more by healthcare professionals. In the FGDs, healthcare professionals mentioned that helping patients with multiple physical complaints was harder as it was unclear what caused what and how to best address complaints.

##### Significant others

Interestingly, significant others ranked topics connected to social life and how patients are approached by others higher than patients. These included ‘invisibility […]’, ‘misunderstanding from outsiders due to limitations’ and ‘awareness of rheumatic diseases and their consequences among the Dutch public’. During the FGDs, these topics were also prominent. Perhaps significant others rank them higher because they themselves also experience (the consequences of) these challenges.

## 4 DISCUSSION

Despite vast improvements in rheumatic disease care since the mid‐twentieth century, we found that the disease still has a considerable impact on the daily lives of many patients. Physical complaints were prioritised most by patients – mainly fatigue and pain. Previous studies have found that pain is constant and impactful for many patients (Erwin et al., 2018; Hunter & Riordan, 2014). Fatigue has also been recognized as an important issue (de Wit et al., 2013) and is increasingly understood to be a complex phenomenon connected to elements such as sleep and biological and psychosocial factors (Davies et al., 2021). Our research adds to this knowledge by elucidating the impact these physical challenges have on mental health, social life and work and education and their complex relationship. It also highlights how many patients desire support to cope with these impacts (Hamnes et al., 2011).

Across patient groups and patient FGDs, challenges were similarly prioritised. However, our results also show heterogeneity within the patient groups, as prioritisation scores were often evenly distributed amongst topics and themes. Based on our qualitative results, we hypothesise that the types of challenges that patients experience or prioritise may as well depend on other factors, for example life‐ and disease phase. Actors looking to address the challenges, should recognise challenges and priorities may vary between individuals. For example, only a fraction of patients prioritised ‘*work and education*’; perhaps those doing so were younger people, who represented only a small proportion of survey respondents. While most of the Dutch rheumatic disease population is of higher age, it is important to acknowledge that younger patients may have different priorities.

Our study highlights that patients' and professionals' priorities can be quite different, but it also indicates that including both actor groups in a mixed‐methods study helps to identify why this is. By mapping the relationships between the experienced challenges of various actors, we were able to better understand the challenges. Patients, for example, expressed frustration that they did not receive sufficient support or care to cope. Healthcare professionals expanded on why referrals to other experts and providing advice are currently difficult. Critical analysis of their different perspectives further explained the causes of challenges, that is there were different views on whether healthcare professionals should have specific rheumatic disease expertise. Self‐management was ranked highly by healthcare professionals. Our FGD results allowed us to hypothesise that this was perhaps because of their slightly different interpretation of the term ‘self‐management’. This shows our mixed‐methods studies' added value, compared with previous studies that explored patients' and healthcare professionals' challenges only qualitatively or quantitatively (e.g. Hunter & Riordan, 2014; Ingegnoli et al., 2020).

A hierarchy can be observed in the survey results, with root causes being prioritised higher than consequences. For example, *physical challenges* give rise to challenges in social life and work and education. Patients may therefore have ranked physical challenges higher in the survey. Another example is the high prioritisation of scientific insights into the impact of lifestyle in *self‐management* and the low prioritisation of prevention and lifestyle advice in *information and options in healthcare*. The FGD results indicate that for prevention and lifestyle advice to be improved, more scientific knowledge is needed.

### 4.1 Strengths and limitations

A strength of the research is the use of the mixed‐methods approach, which allows for better interpretation of both the qualitative and the quantitative data. We argue that quantitative results should be seen jointly with qualitative results: using the survey data in isolation would risk oversimplifying the challenges. Recruiting patients for phase 1 proved quite difficult, and of 59 who enlisted, only 21 eventually participated. We hypothesise that online participation lowers the cancelation and no‐show threshold. Those who participate may do this motivated by a higher disease burden, which may have affected our results. In addition, only a small number of healthcare professionals responded to our FGD and survey invitations. As a result, our ability to study possible varieties of perspectives within various groups of healthcare professionals has been limited. An important shortcoming of our research is that due to the inductive approach, that is, using the FGD results as the input for the survey, stiffness and difficulty moving were initially not identified as separate challenges, and therefore did not occur in the survey as topics. Previous research has reported on these challenges (i.e. Radawski et al., 2019) and indicates that they probably have a high priority for patients. Interestingly, while many respondents reached out to the authors to comment on the survey, none of the 1802 respondents have made comments about these topics missing.

## 5 CONCLUSION

We conclude that our cross‐domain, context‐rich, and multi‐actor study on challenges faced by people with rheumatic diseases can provide a starting point for various actors who wish to take action to improve the lives of this group. Any solutions need to take into account the complexity of these challenges.

## ETHICS STATEMENT

According to Dutch law, the ethical approval of a formal medical ethics committee was not required because this study was non‐invasive and participants were aged over 18. The research complied with the national Code of Ethics for Research in the Social and Behavioural Sciences involving Human Participants (VCWE, 2018). All respondents received written and verbal information about the aim, data analysis and later process of the study and were told that they could withdraw from the study at any time without explanation. All data were anonymised before being analysed.

## AUTHOR CONTRIBUTIONS

Simone Harmsen, Joyce Nabuurs, Marjoleine van der Meij and Carina Pittens were involved in the design, conceptualization and execution of the research project (design, data collection, data analysis). Lotte Lehman de Lehnsfeld helped with the construction of the survey, and conducted the quantitative analysis. Jacqueline Broerse aided in the drafting and pre‐submission revisions of the manuscript, and made conceptual contributions to the publication. Acquisition was done by Carina Pittens, who was also the project leader. She made conceptual contributions to the publication and aided in drafting and pre‐submission revisions of the manuscript. All authors contributed to the writing of the final manuscript.

## CONFLICT OF INTEREST

The authors have no conflict of interest todeclare.

## Supporting information

Figure S1

## Data Availability

Research data are not shared.

## APPENDIX A Survey design, translated from Dutch

**Part 1 Respondent demographics.**

*Questions for all respondents*

- What is your relationship with the Dutch Arthritis Society or rheumatic disease?
  - I myself have a rheumatic disease
  - As a relative of someone with a rheumatic disease
  - As a care or aid provider in primary care
  - As a care or help provider in secondary or primary care
  - As a researcher in the field of rheumatic disease
  - As a technology developer in the field of rheumatic disease
  - As a pharmaceutical developer in the field of rheumatic disease
  - As a volunteer for the Dutch Arthritis Society
  - As a donor of the Dutch Arthritis Society
  - As an investor of the Dutch Arthritis Society
  - As a representative in the field of work or education
  - Other: _____
- What is your age?
  - 18–25 years
  - 25–35 years
  - 35–50 years
  - 50–65 years
  - 65–80 years
  - > 80 years
- Wat is your gender?
  - Male
  - Female
  - Other
- In which province do you live?
  - Noord‐Holland
  - Zuid‐Holland
  - Zeeland
  - Noord‐Brabant
  - Utrecht
  - Flevoland
  - Friesland
  - Groningen
  - Drenthe
  - Overijssel
  - Gelderland
  - Limburg
- What is your highest attained level of education?
  - Primary eduction
  - Vbo/mvo/vmbo/mbo1
  - Havo/vwo/mbo two to four
  - Hbo
  - University

*Questions for specific respondent groups*

**For patients and significant others**

- Please indicate which type(s) of rheumatic disease you have been diagnosed with? Or your significant other has been diagnosed with.
  - Osteoarthritis
  - Axial Spondylarthritis
  - Fibromyalgia
  - JIA (Juvenile Idiopathic Arthritis)
  - Gout
  - Lupus erythematosus (SLE)
  - Lyme disease
  - Osteoporosis
  - Polymyalgia rheumatica
  - Rheumatoid arthritis (RA)
  - Scleroderma
  - Sjogren's syndrome
  - Tietze syndrome
  - A different type, namely: …
  - I don't know
  - I would rather not say
- How long ago have you (or has your significant other) been diagnosed?
  - Less than a year ago
  - Between 1 and 3 years ago
  - Between 3 and 5 years ago
  - Between 5 and 10 years ago
  - More than 10 years ago

**For healthcare professionals:**

- Please indicate which profession you practice:
  - Rheumatologist
  - Child rheumatologist
  - Orthopaedist
  - Dermatologist
  - Rehabilitation doctor
  - Occupational physician
  - Rheumatology nurse or nurse counsellor
  - Pharmacist
  - General Practitioner
  - Physical therapist
  - Other therapists, such as ‘*ergotherapeut’*
  - Sports coach
  - Other, namely: …

**For researchers**

- Please indicate in what field you conduct research:
  - Fundamental research
  - Translational research
  - Clinical research
  - Applied research
  - Other, namely: …

**For volunteers/donors/investors**

- How long have you been connected to the Dutch Arthritis Society?
  - Less than a year ago
  - Between 1 – 3 years ago
  - Between 3 – 5 years ago
  - Between 5 –105 years ago
  - More than 10 years ago

You have now reached the end of the first part of the questionnaire. You can either proceed to the second part of the questionnaire or complete the questionnaire now.

You can click send if you want to complete the questionnaire.

You can click next. Then you will proceed to the second part of the questionnaire. In this section, you are asked to prioritise challenges regarding living with rheumatic disease and opportunities for research and care (which do you think are most important?). This part will take about 10–15 min.

**Part 2 Challenges**

Discussions with patients, significant others, care professionals and other (care) organisations have revealed various challenges and needs that people with a rheumatic disease experience in daily life. These challenges are divided into 6 themes. We ask you to indicate your top two per theme: which challenges do you think should receive the most attention?

It concerns the following six themes:

1. Physical complaints
2. Self‐management
3. Information and options in healthcare
4. Collaboration in healthcare
5. Social and mental wellbeing
6. Work and education

**Prioritisation of topics within themes.**

For each theme the following was asked, after which a list of topics appeared:

Within the theme [THEME NAME] a number of topics have been mentioned as a challenge and/or need.

Please indicate a top 2 of topics, within the theme [THEME NAME], that you think should receive the most attention. Left of the topic, you can enter a number 1 and 2. In the top 2 you enter, you regard the topic for which you fill in a 1, the most important.

**Within the theme [THEME NAME], more attention should be paid to the challenges…**

[The following challenges were presented, for each theme]

**Table:**

**1 Theme: Physical challenges**	
	a. Fatigue
	b. Pain
	c. Invisibility of many rheumatic diseases and complaints
	d. Unpredictability of complaints
	e. Presence of several physical complaints
**2 Theme: Collaboration in healthcare**	
	a. (Local) network of primary and secondary healthcare workers
	b. Acknowledging the experiential expertise of patients
	c. Findability of specialised primary healthcare workers with expertise in rheumatic diseases
	d. Patients often feel they are not being taken seriously by doctors or that doctors think they know what is best for the patients
	e. The control of patients over their own healthcare
**3 Theme: Social and mental wellbeing**	
	a. Constant search for balance in life: between ‘doing’ and ‘not doing’ and between things ‘I have to do’ and things ‘I like to do’
	b. Acceptance of rheumatic disease and its influence on life
	c. Misunderstanding from outsiders due to limitations
	d. The uncertainty a rheumatic disease can cause (i.e. due to a lack of information and delayed diagnosis)
	e. Awareness of rheumatic diseases and their consequences among the Dutch public
	f. (mental) impact of rheumatic diseases on close significant others
	g. Struggling to find meaning in life, because of lack/loss of work and inability to do other daily activities
	h. Shame and taboo around having a rheumatic disease
	i. Effects of rheumatic diseases on intimacy and sexual relations
**4 Theme: Self‐management**	
	a. Influence of lifestyle on several rheumatic diseases
	b. Information on factors relating to effective lifestyle changes, such as nutrition and correct movement
	c. Actively supporting patients to optimise their lifestyle
	d. Actively supporting patients in living their everyday life with a rheumatic disease, for example, scheduling of activities and mental support
	e. Information regarding different rheumatic diseases and treatments
**5 Theme: Information and options in healthcare**	
	a. Correct and fast diagnosis of rheumatic diseases
	b. Reimbursement of primary healthcare costs such as fees for physiotherapists, nutrition coaches and psychologists
	c. More holistic and multidisciplinary healthcare
	d. Mental health and support in learning to live with the rheumatic disease
	e. Side‐effects of medication
	f. Alternative treatments such as homoeopathy, acupuncture and orthomolecular therapy
	g. Prevention
	h. Lifestyle interventions
**6 Theme: Work and education**	
	a. Undesired inability to work
	b. Attitude and flexibility of employers/educational institutes; thinking in terms of possibilities
	c. Communication between patients and employers/supervisors regarding their rheumatic disease, what it is possible for them to do and their needs regarding work
	d. Earning less or not enough as a result of the rheumatic disease
	e. Misunderstanding of colleagues or peers because of disabilities
	f. Difficulties finding a job

## APPENDIX B

Detailed characteristics of survey respondents.

**Table:**

	Sum (N)	Percentage of total (%)	Age in years (N)						Gender (N)	
			18‐25	25‐35	35‐50	50‐65	65‐80	>80	Male	Female
All respondents	1802	100	26	98	352	822	476	28	330	1469
Patients	1503	83,4	24	77	254	705	420	23	216	1285
		Percentage of total patients (%)								
Osteoarthritis	771	51.3								
Axial spondyloarthritis	225	15.0								
Fibromyalgia	437	29.1								
Juvenile idiopathic arthritis	28	1.9								
Gout	35	2.3
Lupus erythematosus	27	1.8								
Lyme disease	9	0.6
Osteoporosis	93	6.2								
Polymyalgia rheumatica	45	3.0								
Rheumatoid arthritis	597	39.7								
Scleroderma	33	2.2								
Sjogren's syndrome	77	5.1								
Tietze syndrome	78	5.2								
Other	93	6.2								
I don't know	11	0.7								
I'd rather not say	2	0.1								
Primary, secondary & tertiary healthcare professionals	79	4.4	0	5	35	35	4	0	21	58
Rheumatologist	12									
Child rheumatologist	1									
Orthopaedic doctor	1									
Rheumatology nurse	28									
General practitioner	2									
Physiotherapist	16									
Other therapist (such as occupation therapist)	4									
Other healthcare profession	17									
Significant others of people (a) with rheumatic condition(s)	54	3,0	1	2	19	14	16	2	20	34
Others	166	9,2	1	14	44	68	36	3	73	92
Researchers	48									
Technology developers	6									
Pharmacists	6									
Volunteers	14									
Donators	24									
Investors	1									
Representatives	7									
Other	60									
