# Supplementary figures and images for: Mapping the complex everyday challenges and needs of people with rheumatic disease and their surroundings using a multi‐actor approach
Source: Musculoskeletal Care. 2022 Apr 27;20(4):873–91. doi: 10.1002/msc.1639 (PMC10084345; doi:10.1002/msc.1639)

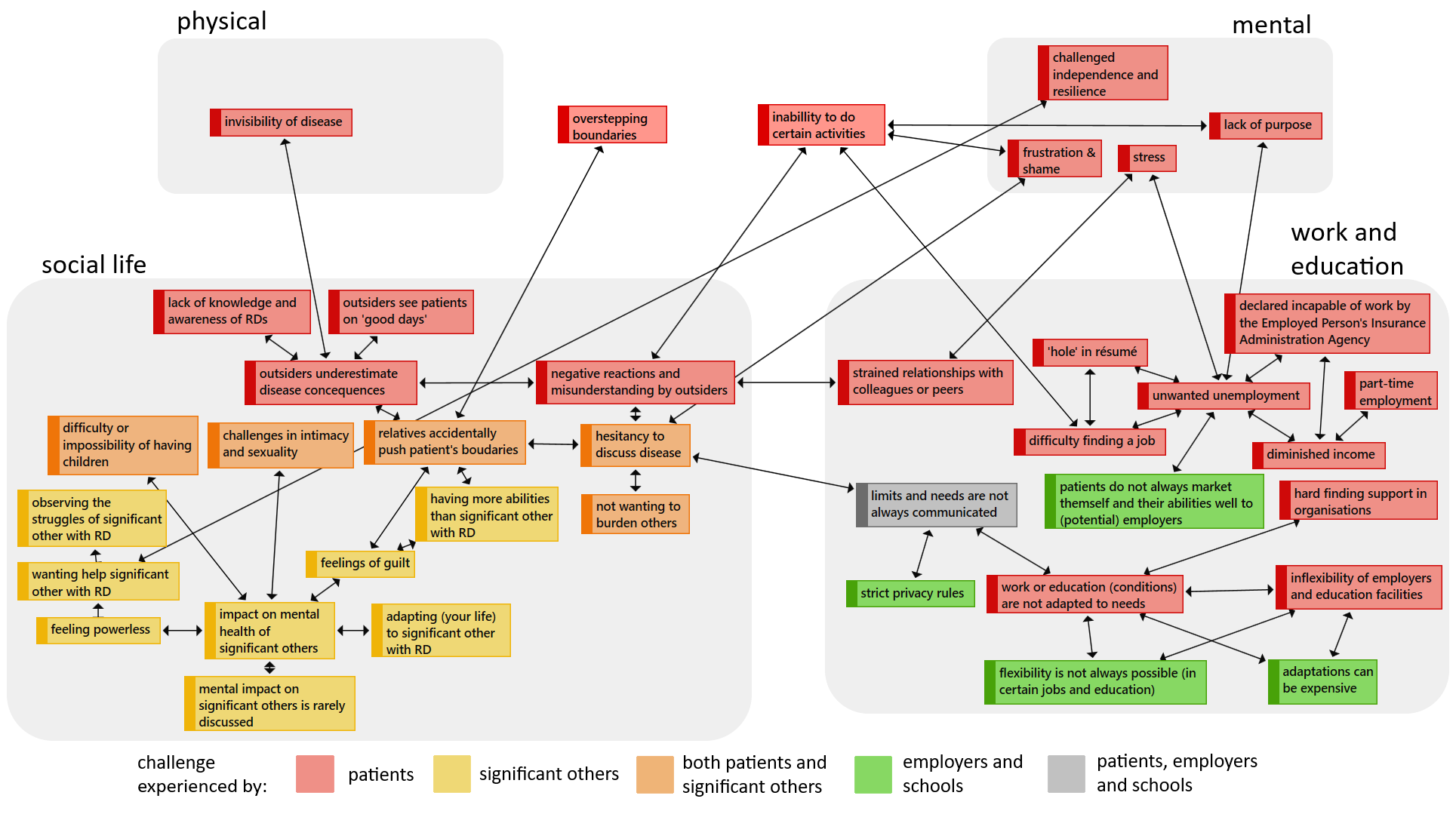

Supplement: Supplementary file 1 — Figure S1 [file MSC-20-873-s001.png]
